# Selenoprotein K Increases Efficiency of DHHC6 Catalyzed Protein Palmitoylation by Stabilizing the Acyl-DHHC6 Intermediate

**DOI:** 10.3390/antiox7010004

**Published:** 2017-12-29

**Authors:** Gregory J. Fredericks, FuKun W. Hoffmann, Robert J. Hondal, Sharon Rozovsky, Johann Urschitz, Peter R. Hoffmann

**Affiliations:** 1 Department of Cell and Molecular Biology, John A. Burns School of Medicine, University of Hawaii, 651 Ilalo Street, Honolulu, HI 96813, USA; gjf@hawaii.edu (G.J.F.); fukun@hawaii.edu (F.W.H.); 2 Department of Biochemistry, University of Vermont, 89 Beaumont Ave, Given Building Room B413, Burlington, VT 05405, USA; robert.s.hondal@gmail.com; 3 Department of Chemistry and Biochemistry, University of Delaware, 136 Brown Laboratory, Newark, DE 19716, USA; rozovsky@udel.edu; 4 Department of Anatomy, Biochemistry and Physiology, John A. Burns School of Medicine, University of Hawaii, 651 Ilalo Street, Honolulu, HI 96813, USA; johann@hawaii.edu

**Keywords:** palmitic acid, palmitoyl-CoA, palmitoylation, inositol 1,4,5-triphosphate receptor, ANK repeat and PH domain-containing protein 2 (ASAP2), cluster of differentiation (CD36), selenium, thioester

## Abstract

Selenoprotein K (SELENOK) is a selenocysteine (Sec)-containing protein localized in the endoplasmic reticulum (ER) membrane where it interacts with the DHHC6 (where single letter symbols represent Asp-His-His-Cys amino acids) enzyme to promote protein acyl transferase (PAT) reactions. PAT reactions involve the DHHC enzymatic capture of palmitate via a thioester bond to cysteine (Cys) residues that form an unstable palmitoyl-DHHC intermediate, followed by transfer of palmitate to Cys residues of target proteins. How SELENOK facilitates this reaction has not been determined. Splenocyte microsomal preparations from wild-type mice versus SELENOK knockout mice were used to establish PAT assays and showed decreased PAT activity (~50%) under conditions of SELENOK deficiency. Using recombinant, soluble versions of DHHC6 along with SELENOK containing Sec_92_, Cys_92_, or alanine (Ala_92_), we evaluated the stability of the acyl-DHHC6 intermediate and its capacity to transfer the palmitate residue to Cys residues on target peptides. Versions of SELENOK containing either Ala or Cys residues in place of Sec were equivalently less effective than Sec at stabilizing the acyl-DHHC6 intermediate or promoting PAT activity. These data suggest that Sec_92_ in SELENOK serves to stabilize the palmitoyl-DHHC6 intermediate by reducing hydrolyzation of the thioester bond until transfer of the palmitoyl group to the Cys residue on the target protein can occur.

## 1. Introduction

Dietary selenium exerts its biological effects mainly through selenoproteins, which all contain the amino acid selenocysteine (Sec) and exhibit a wide variety of functions [1]. The Sec residue has intrinsic redox potential at physiological pH and the selenium within this amino acid side chain is likely to be more resistant to permanent oxidation compared to the sulfur present in the more common amino acid cysteine (Cys) [2] These chemical properties underly the selective advantages for proteins incorporating Sec over Cys [3]. Many selenoproteins exhibit oxidoreductase activity and serve as cellular antioxidants, regulators of redox tone, reducers of oxidized methionine residues, and deiodinases that regulate thyroid hormone [4]. However, some selenoproteins may not function as enzymes and exactly what their biological roles are remains unclear.

We recently identified a new role for one member of the selenoprotein family, selenoprotein K (SELENOK), which appears to be involved in acyl transferase reactions. In particular, SELENOK was shown to interact with the enzyme, DHHC6 (where single letter symbols represent Asp-His-His-Cys amino acids), in the membrane of the endoplasmic reticulum (ER) to promote protein palmitoylation [5]. DHHC enzymes are protein acyl transferases (PATs) conserved throughout eukaryotes that catalyze the addition of acyl groups (e.g., palmitate) onto cysteine residues of substrate proteins in a process termed S-acylation (a.k.a. palmitoylation) [6]. The addition of the 16-carbon fatty acid, palmitic acid, to the sulfhydryl group of cysteine residues forms a thioester bond resulting in S-acylation of the target protein [7]. SELENOK knockout mice are healthy and fertile, although certain aspects of the immune system are compromised [8].

The enzymes contain the conserved 51 amino acid catalytic domain including a DHHC motif embedded within a cysteine-rich domain [9]. DHHC6 is one of 23 members of this family in humans, but it is the only member that contains a Src-homology 3 (SH3) domain. SELENOK is the only selenoprotein with an SH3 binding element, and our previous work showed that interactions via the SH3/SH3 binding domains are essential for DHCC6 and SELENOK binding and subsequent palmitoylation [5]. The DHHC6/SELENOK complex is required for the palmitoylation of certain proteins such as the inositol 1,4,5-triphosphate receptor (IP3R) [5], ANK repeat and PH domain-containing protein 2 (ASAP2) [10], the oxidized low density lipoprotein (LDL) receptor CD36 [11], and likely other proteins [12]. SELENOK appears to serve as a cofactor for the DHHC6 catalyzed S-acylation reaction, but its specific role in facilitating S-acylation has not yet been determined.

DHHC-catalyzed palmitoylation reactions proceed via a two-step ping-pong mechanism [13]. In the first autopalmitoylation step, a Cys residue within the PAT (likely the conserved Cys within the DHHC catalytic domain) reacts with palmitoyl-CoA to form a palmitoyl-PAT intermediate. In the second step, the palmitoyl group is transferred from the PAT to a Cys residue on the target protein. The first step is rapid, whereas the second is much slower. Thus, after the first step, the unstable thioester bond between Cys on the PAT and the palmitic acid may hydrolyze before transfer of the palmitic acid to a target protein, termed the “futile cycle” [14]. This raises the possibility that SELENOK may function to stabilize the palmitoyl-DHHC6 intermediate thereby reducing hydrolyzation of the thioester bond until transfer of the palmitoyl group to the Cys residue on the target protein can occur. In fact, in yeast such stabilization of a palmitoyl-PAT intermediate by the Ethylene-Responsive Transcription Factor 4 (Erf4) cofactor (γ-Tubulin Complex, GCP16, in mammals) has been shown for the Erf2 PAT (DHHC9 in mammals) [15,16]. There are also intriguing questions of whether Cys instead of Sec in SELENOK would be able to equivalently function to facilitate DHHC6 catalyzed palmitoylation. With these questions in mind, we set out to develop in vitro assays to study molecular mechanisms of S-acylation by the DHHC6/SELENOK complex.

## 2. Materials and Methods

### 2.1. Reagents and Mice

Ni-NTA agarose for cytosolic DHHC6 purification from bacteria was purchased from Qiagen (Hilden, Germany). Dynabeads for His-tag isolation and pull-down of DHHC6 during subsequent assays were obtained from ThermoFischer (Waltham, MA, USA). All versions of SELENOK with an N-terminal 8 amino acid StrepII tag (WSHPQFEK) were expressed in bacteria and purified in the laboratory of Dr. Sharon Rozovsky at the University of Delaware as previously described [16,17]. StrepTactin™ beads (Millipore-Sigma, Burlington, MA, USA) were used for SELENOK pull-down. The FITC-labeled fluorescent peptide corresponding to the amino acid sequence surrounding the N-terminal dicysteine palmitoylation consensus site of CD36 (MGCDRNCK) was purchased from Peptide 2.0 (Chantilly, VA, USA). Palmitoyl-CoA (C16:0) lithium was purchased from Sigma (St. Louis, MO, USA) and NBD-palmitoyl-CoA purchased from Avanti Polar Lipids. Antibodies used to detect DHHC6 and SELENOK were previously described [5]. C57BL/6J wild-type controls were generated from mice originally purchased from the Jackson Laboratory (Bar Harbor, ME, USA). Generation of SELENOK KO mice was previously described [8]. All animal protocols were approved by the University of Hawaii Institutional Animal Care and Use Committee (Animal Welfare Assurance number: A3423-01).

### 2.2. DHHC6 Preparation

We generated a soluble cytosolic DHHC6 enzyme by cloning the NCBI sequence NM_001033573.1 encoding the two functional cytosolic domains of DHHC6 connected by a flexible linker domain (GGGSGGGSGGGS) into a pET-14b His-tagged expression vector for bacterial expression (GenScript, Piscataway, NJ, USA). His-tagged cytosolic DHHC6 was expressed in *E. coli* BL21(DE3) cells, purified using a Ni-NTA agarose column and stored in aliquots at −80 °C until they were ready to be used. Prior to running the assay, aliquots of cytosolic DHHC6 were thawed and incubated overnight in 50 mM MES, pH 6.4 containing 0.05% DDM and 10 mM DTT.

### 2.3. Polyacrylamide Based Analyses of S-Acylation of DHHC6

For studies examining the influence of SELENOK on cytosolic DHHC6 autoacylation, His-tagged DHHC6 protein was incubated overnight at a 1:1 molar ratio with three versions of SELENOK (U92A, U92C and U92) that each included an N-terminal streptavidin-tag II as well as 2 mg StrepTactin™ beads (Millipore, Burlington, MA, USA) in 50 mM MES, pH 6.4 containing 0.05% DDM and 10 mM DTT. The following day, beads were washed three times and incubated in 50 mM MES (pH 6.8 or 7.4 or 8.2) containing 0.05% DDM and 10 μM NBD-palmitoyl-CoA at 30°C for 60 min with aliquots collected from the magnetic beads. The beads were re-suspended in sample loading buffer (50 mM Tris–HCl (pH 6.4), 2% SDS, 12.5 mM EDTA, 10% glycerol) and subjected to 10–20% Tris-Tricine SDS–PAGE. Fluorescent bands in the gel were visualized using a UV gel box with DHHC6 acylation represented by fluorescent NBD-palmitoyl-DHHC6. The gel was then stained using Coomassie Brilliant Blue (Thermo Fisher Scientific, Waltham, MA, USA) per manufacturer’s instructions to validate equivalent quantities of SELENOK as well as equivalent DHHC6 pull-down. Western blots were performed and analyzed using an Odyssey scanner (Li-Cor, Lincoln, NE, USA) as previously described [18].

### 2.4. TLC-Based Fluorescent Peptide Microsomal PAT Assay

Spleens were collected from WT and SELENOK KO mice and leukocytes separated as previously described [19], and ER microsomes isolated from splenocytes for use in the PAT assay. In brief, spleens were homogenized into a single cell suspension by passage through a 100 μm filter followed by lysis of the red blood population using ammonium chloride based red blood cell lysis buffer (Sigma, St. Louis, MO, USA). Intact white blood cells (2 × 10^8^ per condition) were separated from red blood cell debris via centrifugation and subsequently re-suspended in isotonic extraction buffer (50 mM HEPES, pH 7.8, with 1.25 M sucrose, 5 mM EGTA, and 125 mM potassium chloride containing protease inhibitor cocktail (Millipore-Sigma, Burlington, MA, USA) and lysed by sonication. The post-mitochondrial supernatant was obtained via a low-speed centrifugation (12,000× *g*) and was subsequently centrifuged at 100,000× *g* for one hour to obtain the ER microsomal pellet. This pellet was re-suspended in 50 mm MES (pH 6.4) buffer containing 0.05% DDM at a ratio of 10 mg ER microsomes per 1 mL of buffer. Prior to PAT assay, FITC-labeled MGCDRNCK peptide (1 mM, 100× stock) was treated with 10 mM DTT at room temperature for 1 h to reduce disulfide-linked peptide dimers. To initiate reactions, the reduced peptide was added to the aqueous reaction buffer containing the ER microsomes to yield a final concentration of 10 μM peptide in 0.05% DDM, 0.1 mm DTT and 50 mm MES, pH 6.4, and reactions were incubated at 30 °C for 2 to 60 min. After reaction completion, 10 μL of reaction volume was spotted on reverse-phase C18 TLC plates uniplates (Analtech, Newark, DE, USA) per time point and resolved using a 40% acetonitrile mobile phase. Post-run fluorescent peptides were visualized under UV fluorescent light to determine the ratio of palmitoylated to non-palmitoylated peptide. For TLC evaluation of the cytosolic DHHC6/SELENOK complexes, a similar protocol was followed with modifications. As described above, the intermediates generated by incubating cytosolic DHHC6, soluble SELENOK (U92 → A, U92 → C, U92), and NBD-palmitoyl-CoA at pH 7.4 were washed in the same buffer and incubated with the CD36 peptide in 0.05% DDM, 0.1 mm DTT and 50 mm MES, pH 6.4, and reactions carried out at 30 °C for 2 to 15 min. Thin layr chromatography (TLC) was carried out as described above.

## 3. Results

### 3.1. SELENOK Deficiency Leads to Decreased Target Peptide Palmitoylation Using an In Vitro Assay

**Figure 1:**
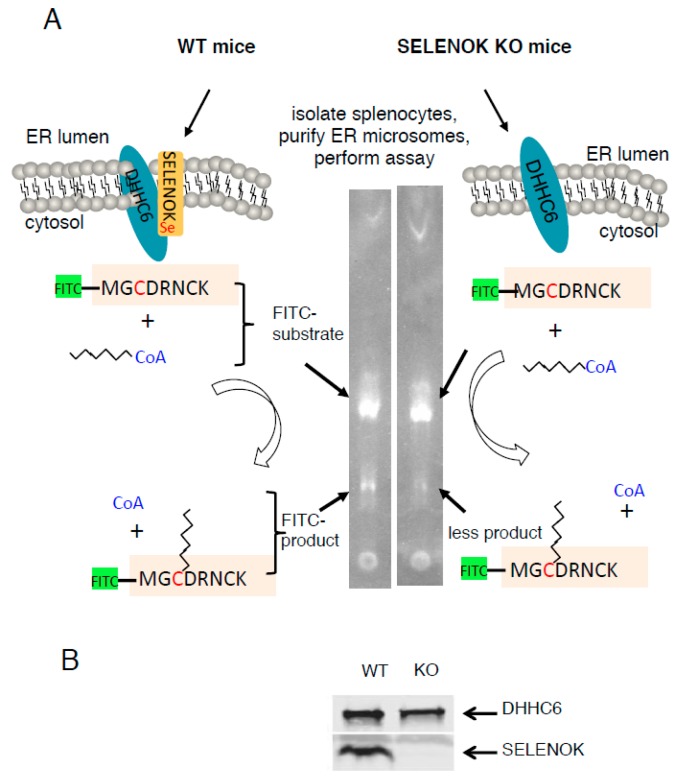
A diagram illustrating protocol for detecting palmitoylation of peptides by murine splenocyte microsomes. (**A**) Splenocytes were isolated from either WT or SELENOK mice, and microsomes were prepared containing ER membranes with or without SELENOK, respectively. Substrates including palmitoyl-CoA and FITC-peptide were added to microsomes for different periods of time after which the reaction mixtures were subjected to reverse-phase TLC. Fluorescent bands corresponding to palmitoylated peptides were identified using UV transillumination. (**B**) For each experiment, equivalent levels of DHHC6 were confirmed using Western blotting.

**Figure 2:**
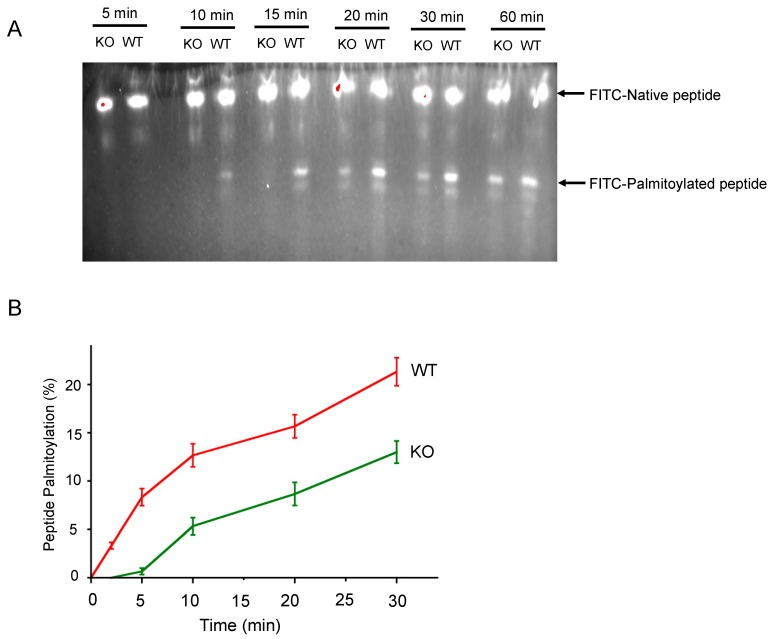
Palmitoylation of target peptides is decreased in SELENOK deficient microsomes. (**A**) A time course for peptide palmitoylation was performed using a TLC-based assay. A representative image for fluorescence imaged with a *uv* transilluminator. Note the imasge was was captured at high exposure to clearly show the palmitoylated peptide bands, which resulted in some red overexposed regions in the native peptide band. (**B**) Three independent experiments were conducted to compare microsomes from WT and SELENOK KO splenocytes. Values at each time point indicate percent of peptide that was palmitoylated = (FITC/(Native + FITC)) × 100. Means ± S.E. are shown.

We set out to test the requirement of SELENOK for S-acylation (i.e., palmitoylation) of a target peptide by the DHHC6 in the ER membrane. To this end, we purified ER microsomes from splenocytes of WT or SELENOK KO mice and tested their capacity to palmitoylate a peptide from a known palmitoylation target of DHHC6/SELENOK, CD36 [10]. Using thin layer chromatography to differentiate between the native and S-acylated forms of a FITC-labeled CD36 peptide (MGCDRNCK), we found that ER microsomal preparations from SELENOK KO splenocytes exhibited lower levels of palmitoylation compared to WT controls (Figure 1A). Western blotting confirmed that this was not due to differences in enzyme levels (DHHC6) in the microsomal preparations (Figure 1B). These experiments were repeated using a time course approach and SELENOK deficient ER microsomes were found to generate approximately 50% less S-acylated peptide over time (Figure 2). These results are consistent with in vivo data in WT versus SELENOK KO mice and validated our ability to utilize TLC-based palmitoylation approaches to evaluate molecular features of SELENOK facilitated DHHC6 catalyzed alkylation.

### 3.2. The Sec Residue in SELENOK Facilitates S-Acylation of DHHC6

**Figure 3:**
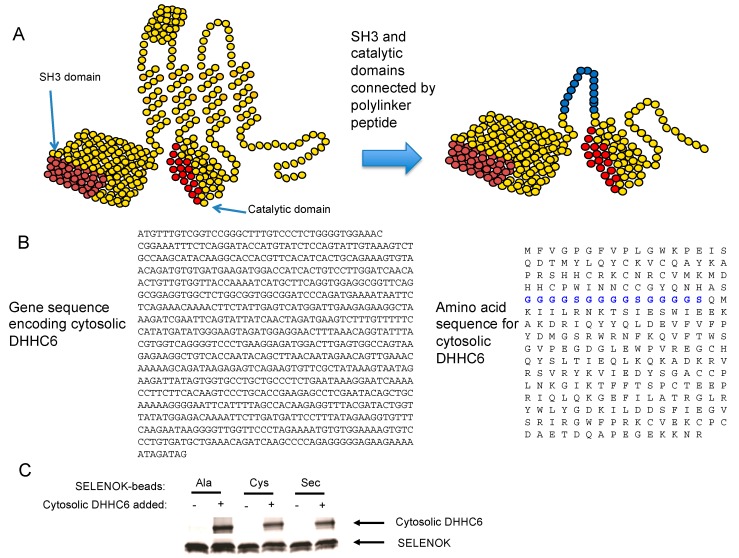
Synthesis of a soluble cytosolic DHHC6 that binds to SELENOK. (**A**) Illustration of full-length DHHC6 with the SH3 and catalytic domains that were used to construct a modified version of DHHC6, which contains both domains connected by a linker domain shown in blue. (**B**) The DNA sequence used to express the cytosolic DHHC6 along with amino acid sequence showing linker region in blue. (**C**) StrepTactin beads were used to pull down 1 μg streptavidin-tagged SELENOK containing either Ala, or Cys, or Sec at amino acid position 92. These beads were divided into two aliquots that were not or were incubated overnight at 4 °C with the cytosolic DHHC6 in a 1:1 molar ratio. Eluted proteins were analyzed by Coomassie blue staining of PAGE, and results show all three versions of SELENOK bound to cytosolic DHHC6. Note that some degradation of the recombinant SELENOK occurred during the overnight incubation giving rise to double bands. Also, these are recombinant proteins and not cell lysates, which leads to no detectable background in lanes corresponding to the “no Cytosolic DHHC6 added” conditions.

In order to study mechanisms by which SELENOK influences the catalytic activity of DHHC6, we cloned and expressed a soluble version of DHHC6 that contains the SH3 domain to bind to SELENOK and catalytic domain (Figure 3A,B). A flexible linker domain that has been used in previous studies to successfully link protein domains in a similar fashion [20] was used to link these two domains of DHHC6. This cytosolic DHHC6 protein was incubated with variants of full-length SELENOK with different amino acids at the active position of amino acid 92 (U92 → A, U92 → C and U92) that were coupled to StrepTactin™ beads via an N-terminal Strep-II tag. We were able to bind equivalent quantities of cytosolic DHHC6 to all three version of bead-bound SELENOK (Figure 3C).

**Figure 4:**
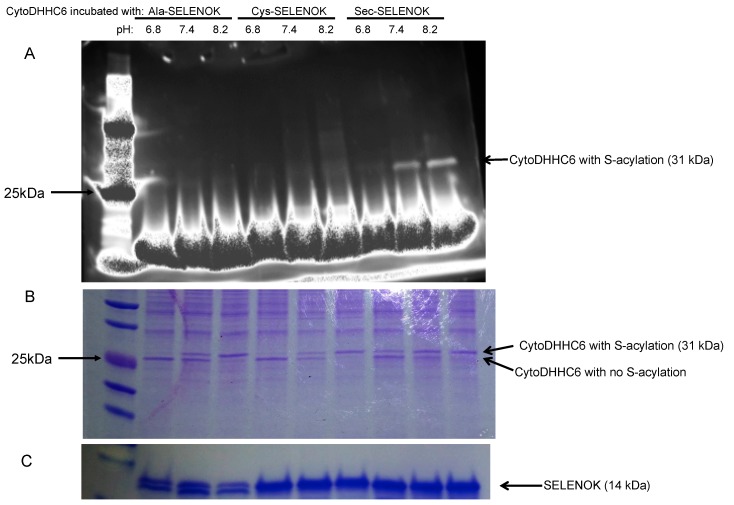
Sec-containing SELENOK stabilizes acyl-DHHC6 intermediate formed during the autopalmitoylation of the enzyme. Cytosolic DHHC6 bound to different versions of SELENOK (U92 → A, U92 → C, and U92) bound to StrepTactin beads were incubated for 60 min with fluorescent NBD-palmitoyl-CoA in buffer of pH 6.8, 7.4, and 8.2. Beads were then washed, proteins eluted and analyzed by SDS-PAGE. (**A**) The gel was exposed to *uv* using a transilluminator and the acylated DHHC6 visualized at ~31 kDa with highest levels found in the lanes corresponding to Sec-containing SELENOK at pH 7.4 and 8.2. (**B**) Coomassie Blue staining of the gel revealed two bands for DHHC6 ~31 kDa. Based on the pattern within the pH ranges, the upper band corresponds to acylated DHHC6 and the lower band to the nonacylated DHHC6. (**C**) Coomassie Blue staining of the gel in the region of SELENOK (~14 kDa) shows that the protein was eluted from beads at equivalent levels. Note that some degradation of the recombinant SELENOK occurred during the overnight incubation giving rise to double bands.

These different DHHC6/SELENOK complexes were then incubated with a fluorescent version of palmitoyl-CoA (NBD-palmitoyl-CoA) to allow autoacylation of DHHC6 to occur at different pH. Autoacylation has been shown to exhibit pH dependence, with acylated DHHC enzymes hydrolyzing at low pH (<7.2) but maintaining acylated states at higher pH (between 7.4 and 8.6) [13]. In the case of Erf2/Erf4 autopalmitoylation, the underlying cause for the observed pH dependence was proposed to be the charges on key histidines. Thus, to study the stability of the acylated DHHC6 intermediate, autoacylation of DHHC6 at carried out at pH 6.8 (conducive to hydrolysis), pH 7.4 (low levels of hydrolysis), and pH 8.2 (very low levels of hydrolysis). A fluorescent NBD-palmitoyl-CoA was incubated with the bead-bound DHHC6/SELENOK complexes and the autoacylation of DHHC6 was analyzed by SDS-PAGE. The fluorescent detection of acyl-DHHC6 within the gel showed that Sec-containing SELENOK was most effective at generating or stabilizing acylated DHHC6 (Figure 4A). A fluorescent band corresponding to stable acyl-DHHC6 was detectable at both pH 7.4 and 8.2. As a parallel approach, the gel was stained with Coomassie Blue and this allowed visualization of acylated and non-acylated versions of DHHC6 based on size/mobility differences (Figure 4B). The DHHC6 complexed to Ala-containing SELENOK represents baseline autoacylation of the DHHC protein due to the absence of the important Sec residue at position 92 in SELENOK. Under conditions conducive to hydrolysis of the acylated intermediate (pH 6.8), there was no detection of acyl-DHHC6. Autoacylation of DHHC6 complexed to Ala-containing SELENOK increased under conditions less conducive to hydrolysis (pH 7.4) and was fully acylated under conditions non-conducive to hydrolysis (pH 8.2). Cys-containing SELENOK complexed to DHHC6 generated similar results, suggesting that Cys residue at position 92 did not facilitate acylation of DHHC6 above the baseline autoacylation levels. However, Sec-containing SELENOK complexed to DHHC6 led to stable acyl-DHHC6 under conditions conducive to hydrolysis (pH 6.8), and higher levels of acylation at the less conducive conditions (pH 7.4 and 8.2). These data together with the fluorescent data shown above suggest that Sec at amino acid position 92 in SELENOK stabilizes the acyl-DHHC6 intermediate by protecting the thioester bond of acyl-DHHC6 from hydrolysis that leads to the futile cycle.

**Figure 5:**
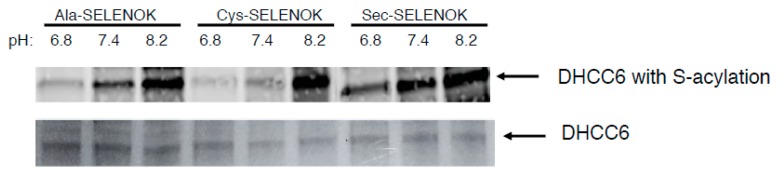
Sec-containing SELENOK is most effective at stabilizing the acyl-DHHC6 intermediate. The autopalmitoylation of cytosolic DHHC6 was performed with different bead-bound versions of SELENOK (U92 → A, U92 → C, and U92) under different pH conditions. Western blotting was performed to detect NBD-palmitate-DHHC6 as a green fluorescent band and the DHHC protein that was detected with anti-DHHC6 and IR-secondary antibodies as a red fluorescent band.

As another approach to study S-acylation of DHHC6, western blotting was used to analyze the different versions of SELENOK for their ability to facilitate S-acylation of DHHC6. As described above, fluorescent NBD-palmitoyl-CoA was incubated with DHHC6 complexed to different versions of SELENOK (U92 → A, U92 → C, U92) at pH 6.8, 7.4, and 8.2. Similar to the results above, complexing of DHHC6 to Sec-containing SELENOK led to stable acyl-DHHC6 at the conditions conducive to hydrolysis (pH 6.8) with S-acylation increasing under conditions less conducive to hydrolysis (pH 7.4 and 8.2) (Figure 5). As discussed above, Ala-containing SELENOK combined to DHHC6 represents autopalmitoylation of DHHC6 in the absence of functional SELENOK. No stable acyl-DHCC6 intermediate was detected under conditions conducive to hydrolysis (pH 6.8), but acylation of DHHC6 increased when pH was increased. Results for Cys-containing SELENOK were similar to Ala-containing SELENOK, suggesting that Sec residue at position 92 is required for any increase in S-acylation of DHHC6 above baseline levels.

### 3.3. The Acyl Transfer Function of DHHC6 is Most Efficient with Sec-Containing SELENOK

**Figure 6:**
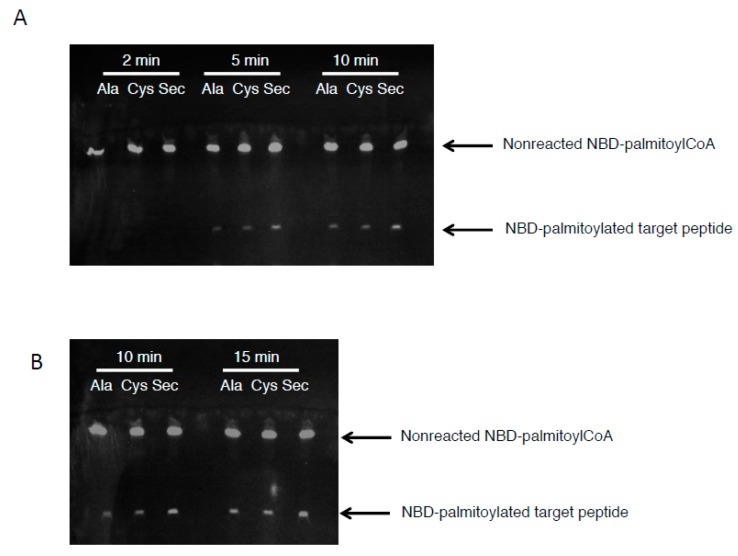
Acyltransferase activity of DHHC6 is most efficient when bound to Sec-containing SELENOK. The NBD-palmitoyl-DHHC6 intermediates formed at pH 7.4 were incubated with CD36 peptide (MGCDRNCK) for different periods of time. Reactions were terminated with SDS on ice and reaction products run on reverse-phase TLC, followed by analysis on a *uv* transilluminator to detect NBD-palmitoyl-peptide. (**A**) The first experiment was run at three time periods of 2, 5, and 10 min. (**B**) The second experiment was run for 10 and 15 min.

While the data above suggest the acyl-DHHC6 intermediate is stabilized by binding to Sec-containing SELENOK, this does not demonstrate the ability of DHHC6 to transfer this acyl group to target peptides. Thus, we next incubated the NBD-palmitoyl-DHHC6 intermediates (formed at pH 7.4) with a target peptide corresponding to CD36 for different periods of time. The transfer of the fluorescent NBD-palmitate was analyzed by thin layer chromatography as described above, and results showed that the acyl transfer at 5 and 10 min was most effective for DHHC6 complexed to Sec-containing SELENOK (Figure 6A). This was repeated for 10 and 15 min and again, at 10 min DHHC6 complexed to Sec-containing SELENOK was most effective at transferring the fluorescent palmitate to the target peptide (Figure 6B). By 15 min the levels of acyl-peptide were equivalent for DHHC6 complexed to Ala-, Cys-, and Sec-containing SELENOK. Thus, both stability of the acyl-DHHC6 intermediate and efficiency of transferring the acyl group to target peptides were increased when DHHC6 enzyme was bound to SELENOK that contained Sec at residue 92.

## 4. Discussion

The data presented herein provide insight into how SELENOK bound to DHHC6 may increase the catalytic efficiency of the S-acylation reaction. Our data show that the Sec residue at position 92 of SELENOK is important for stabilizing the autopalmitoylated intermediate, acyl-DHHC6, to prevent hydrolysis of the thioester bond holding the palmitate to the DHHC6 enzyme. This likely increases the time for the enzyme to “find” its target protein within cells to transfer the palmitate group and complete the reaction. Compared to Ala or Cys, Sec at position 92 in SELENOK allows a faster transfer of the palmitate from DHHC6 to Cys residues on target peptides. In fact, SELENOK with Cys at position 92 did not improve stability of the acyl-DHHC6 intermediate above SELENOK with Ala at the same position, particularly at physiological pH (7.4) or pH conducive to hydrolysis (6.8). Also, Cys was similar to Ala at position 92 in SELENOK in terms of ability to promote acyl transfer from acyl-DHHC6 to target peptide. Of course, these results were obtained from cell-free systems that do not inlcude important aspects of DHHC6 catalyzed palmitoylation reactions within cells. For example, a recent study found that DHHC6 may act in concert with DHHC16 to perform its catalytic role in cells [21], and this will need to be considered when studying the influence of SELENOK on DHHC6.

Palmitoylation occurs by a two-step mechanism as best demonstrated in the yeast DHHC enzyme complex, Erf2/Erf4. In *Saccharomyces cerevisiae*, Erf2/Erf4 is a heterodimeric PAT that palmitoylates yeast Ras2 on Cys-318. In the first step, the Erf2 subunit of this PAT complex undergoes autopalmitoylation to create a thioester-linked palmitoyl intermediate. In the second step, this intermediate either undergoes hydrolysis (futile cycle) or carries out the transfer of palmitate from the enzyme cysteine to the cysteine of the Ras substrate [13]. The Erf4 subunit functions to stabilize the Acyl-Erf2 intermediate and to avoid the futile cycle of hydrolysis. Our data suggest SELENOK functions similar to the Erf4 subunit by stabilizing Acyl-DHHC6 and improves the overall transfer of the palmitate to target peptides. Moreover, the Sec residue at position 92 is key for improving intermediate stability and acyl transferase activity. As stated above, Cys at position 92 did not improve acyl-DHHC6 intermediate stability or acyl transferase activity above those levels found with Ala at the same position. Since Ala-containing SELENOK represents a “functionally inactive” form of SELENOK with no redox amino acid (there are no other Cys present in the protein), it appears a Cys residue at position 92 in SELENOK is unable to contribute to DHHC6 catalytic functions. However, there are caveats to these interpretations. First, these data were obtained using a modified, cytosolic form of DHHC6 bound to bead-bound SELENOK. Since the palmitoylation of target proteins by DHCC6/SELENOK in the cell occurs at the surface of the ER membrane, the differences between Sec and Cys in SELENOK may not be fully characterized by in vitro assays. Also, without definitive kinetic data the precise differences in enzymatic activity cannot be determined. Our future research will be aimed at overcoming these two limitations.

These findings highlight the importance that nature has placed on stabilizing the acylated DHHC intermediates formed during palmitoylation reactions. In addition to the Erf2/Erf4 example given above, evidence is emerging that other DHHC enzymes employ different strategies to obtain stable intermediates. For example, DHHC9 contains a key amino acid (Arg148) in the Cys-rich domain that when mutated leads to increased loss of palmitate from its acylated intermediate through hydrolysis, suggesting this residue is important for preventing water mediated nucleophilic attack [22]. The possibility that SELENOK functions to stabilize the acyl-DHHC6 intermediate and prevent hydrolysis before the acyl group can be transferred to a target protein highlights the importance of this step in the palmitoylation reaction that physiologically occurs on the cytosolic side of the ER membrane. Synthesis of SELENOK requires sufficient dietary selenium and dedicated translational machinery to insert the 21st amino acid into position 92 [1,3,18]. Exactly how SELENOK uses its Sec residue to stabilize the acyl-DHHC6 intermediate was not determined in this study. It may involve acyl-transfer equilibrium between the thioester bond in DHHC6 and a selenol ester bond in SELENOK that prevents hydrolysis of thioester bond by transferring the acyl group to Sec as a selenol ester bond and then back to DHHC. In fact, SELENOK has been shown to form dimers and experimental evidence highlights the reactivity of selenoesters with selenocystines, so there is the possibility that complex thioester (in DHHC6) and selenoester (In one or more SELENOKs) bonds are involved in the catalytic mechanism [16,23].

## 5. Conclusions

Overall, our data suggest increasing the efficiency of DHHC6 catalyzed palmitoylation requires Sec in SELENOK that cannot be replaced by Cys. Without SELENOK present in the immune cells, the stability of the acylated DHHC6 intermediate is compromised leading to reduced palmitoylation of key proteins involved in promoting immune cell functions like proliferation, migration, and cytokine secretion. Thus, these new data provide mechanistic insight into previous findings showing that the SELENOK knockout mice exhibit compromised immunity and our laboratory plans to expand on these cell-free assays as we move forward with further in vitro and in vivo experiments.
